# Effect of different antidiabetic medications on atherosclerotic cardiovascular disease (ASCVD) risk score among patients with type-2 diabetes mellitus: A multicenter non-interventional observational study

**DOI:** 10.1371/journal.pone.0270143

**Published:** 2022-06-28

**Authors:** Syed Wasif Gillani, Syed Azhar Syed Sulaiman, Vineetha Menon, Nazeerullah Rahamathullah, Riham Mohamed Elshafie, Hassaan Anwer Rathore

**Affiliations:** 1 Department of Pharmacy Practice, College of Pharmacy, Gulf Medical University, Ajman, UAE; 2 Advance Medical and Dental Institute (IPPT), Universiti Sains Malaysia (USM), Penang, Malaysia; 3 Department of Biomedical Sciences, College of Medicine, Gulf Medical University, Ajman, UAE; 4 Clinical and Hospital Pharmacy Department, College of Pharmacy, Taibah University, Al Madinah Al Munawwarah, Saudi Arabia; 5 Clinical Pharmacy Department, ASUSH, Ain Shams University, Cairo, Egypt; 6 College of Pharmacy, QU Health, Qatar University, Doha, Qatar; University Magna Graecia of Catanzaro, ITALY

## Abstract

**Objective:**

The aim of this study was to compare the clinical outcomes associated with different combinations of oral diabetic drugs among patients with type 2 diabetes mellitus.

**Method:**

A prospective multicenter longitudinal, noninterventional observation study design was applied. At baseline (0 month), clinical parameters including glucose profile, renal function, lipid profile and risk assessment for cardiovascular risks were calculated. Mean Weighted difference (MWD) with heterogeneity and effect z was calculated to determine the risk reduction at the end of the study.

**Results:**

A total of 1,657 were enrolled to different cohorts with response rate of 75.5%. The distribution of patients was based on prescribed drug. A total of 513 (30.9%) in G1 (metformin alone), 217 (13.09%) in G2 (metformin with Glimepiride), 231 (12.85%) in G3 (Metformin with Gliclazide), 384 (23.17%) in G4 (metformin with Sitagliptin) and 312 (18.89%) in G5 (Metformin with Saxagliptin). There was no significant different in all clinical and social variables at baseline. The Intergroup analysis showed significant differences with all the primary outcome variables except BMI (*p* = 0.217) and eGFR (*p =* 0.782) among patients using sulphonylurea (SU) combination (G2 & G3). Findings also showed significant high frequency of emergency visit and hospitalization in G1 (78.16% & 30.8%) as compared to SU (70.1% & 28.3%, *p =* 0.001) and DPP-4 (56.6% & 20.4%, *p =* 0.001). The overall reported effect was z = 2.58, *p* = 0.001 for ASCVD risk reduction assessment.

**Conclusion:**

The study concluded that significant effect of *Dipeptidyl peptidase*-4 inhibitor on reduction of hospitalization, lipid profile and also ASCVD risk score of type-II diabetes mellitus patients regardless of clinical comorbidities. Also, sulfonylurea combinations have showed significant reduction in LDL and triglycerides values.

## 1. Introduction

Type 2 diabetes is a progressive syndrome associated with short term and long-term complications. Patients with type 2 diabetes mellitus have a high risk for cardiovascular complications like dyslipidemia, hypertension and atherosclerotic cardiovascular events and suffer from substantial morbidity and mortality [1, 2]. Worldwide projections on the prevalence of T2DM reported 70% increase in developing countries and 20% in developed countries [1, 3]. Despite effective monotherapy for diabetes, approximately 50% of patients require additional medications after 3 years to achieve target glycosylated hemoglobin (A1C) < 7% [1, 2].

Due to the progressive nature of the disease, multiple antihyperglycemic drugs like sulfonylureas and metformin are required for most patients to attain and maintain euglycemia status. Each agent offers a unique set of risk and benefit that must be considered on individualized basis [3]. Sulfonylureas stimulate insulin secretion from pancreatic beta cells independent of plasma glucose concentration and metformin is a biguanide that lowers hepatic glucose production with potential increase in insulin sensitivity. DPP-4 inhibitors like sitagliptin stabilizes the incretin peptides glucagon-like peptide-1 (GLP-1) and glucose-dependent insulin tropic peptide, resulting in a glucose-dependent increase in plasma insulin levels and subsequent decrease in plasma glucagon levels [3–5].

Metformin is considered as a first line drug and is efficacious in controlling glucose, insulin sensitizing and body weight effects [1, 3–5]. However, metformin has limited effect for longer duration and necessitates the use of additional drugs. Sulfonylurea (SU) drugs are often added to address this inadequacy. Glycemic control improves with the addition of SU but the efficacy is not persistent due to associated adverse events [1].

Sulfonylureas are associated with weight gain and adverse effects like hypoglycemia, which is more common among elderly than adult patients [3, 4]. Moreover, some drugs increase the hypoglycemic effect of sulfonylureas by protein displacement and reducing hepatic metabolism or decreasing urinary excretion requires extensive drug therapy workup [2, 3]. Treatment with sulfonylureas is also associated with fatal arrhythmias, weight gain, increased risk of QT prolongation, increased cardiovascular events and mortality compared with other glucose-lowering drugs [2–4]. The risk factors for the development of cardiovascular complications are multifactorial including unintended effects of antihyperglycemic medication on ASCVD score, weight gain and/or hypoglycemia [5, 6].

Similarly, third generation sulfonylurea like glimepiride is reported to induce Syndrome of Inappropriate Antidiuretic Hormone Secretion (SIADH) leading to hyponatremia (serum Na < 135 mmol/L) without dehydration, increased renal excretion of sodium (urinary Na > 20 mmol/L), low plasma osmolality (< 280 mOsm/kg) and high urinary osmolality (> 300 mOsm/kg) among patients with normal renal and adrenal function [3, 6, 7].

Other antidiabetic drugs like glimepiride and glipizide are associated with adverse effects like dizziness, syncope, headache, nausea, and increased serum levels of liver enzymes [1, 6]. Some of the rare reported adverse effects include angioedema, shock, agranulocytosis, aplastic anemia, disulfiram-like reaction, hypersensitivity, maculopapular rash, Stevens-Johnson syndrome, cholestatic jaundice, hepatic failure, accommodation disturbance (early during treatment) and others [5, 7]. The use of metformin has relatively low incidence of weight gain or hypoglycemia. The incidence of rare adverse event lactic acidosis is approximately 0.03 cases per 1000 patient, with 0.015 reported fatality and commonly associated with renal failure [3–5, 7]. Dipeptidyl peptidase 4 (DPP-4) inhibitors are newer antidiabetics. Adverse drug events include acute renal failure, erectile dysfunction, peripheral neuropathy, renal insufficiency, and severe arthralgia [7, 9]. Acute pancreatitis are rarely reported in patients after treatment with incretin-based therapies [8].

Evidence-based practice showed intensive glycemic control reduces the development and progression of complications [7, 8]. Several treatment regimens are available to individualize treatment plan for patients with glucose intolerances. Combination therapies are often required for diabetic patients in addition to life-style modifications [8, 9]. Literature reported treatment failure among 50% of newly diagnosed patients treated with monotherapy diabetic therapy [4, 6, 7]. Individualization selection of drug and potency are the key-elements to treatment success and maintaining targeted HbA1c levels [4, 7, 8].

The therapeutic goals in the management of diabetes mellitus often focused to serum glycemic levels, therefore the prevention of cardiovascular events is seldom missed or overlooked [4–7]. Several evidences suggested efficacious role of pioglitazone in the reduction of coronary atherosclerosis compared to glimepiride [2, 3]. However, there are limited studies to support the efficacious preference of oral hyperglycemic drugs towards preferred prescribing in cardiovascular risk reduction [9]. The Food and Drug Administration (FDA) also excluded the approval of antidiabetic drugs without cardiovascular reported risk data [10]. Therefore, the clinical trials are shifted to determine the effect of new antidiabetic drugs on the prevention of cardiovascular events [10].

Majority of the antidiabetic medications are associated with several adverse effects, resulting in poor compliance and subsequent worsening of glucose intolerance. Healthcare providers and patients are required to objectively assess the risk and benefit of antidiabetic agents before developing a individualize care plan. In the present study is aimed to evaluate the clinical outcomes and ASCVD risk score among patients treated with combination of oral antidiabetic either with DPP4 inhibitors or sulfonylureas.

## 2. Methods

### 2.1 Ethical approval

The study was performed in compliance with World medical association (WMA) *declaration of Helsinki*: *Ethical principles for medical research involving human subjects amended by* 59^th^ WMA (number PHRC/HC/11/13), 2013 Seoul, Korea. The study was approved by clinical research committee (CRC) 2017, Ministry of Health (MOH), Malaysia (NMRR-10-776-6941). The study protocol and follow-up procedure followed the Good Clinical Practice (GCP) guidelines 2017, MOH, Malaysia.

Patients agreed to join the study were require to sign a research informed consent form. Patients, who had difficulty in reading or understanding, acquire an impartial witness to explain the study protocol and follow-up procedures before participation. Patients were briefed extensively about the dropout criteria. Patients were also briefed about the voluntarily participation and leaving the study at time will not affect the usual care process.

### 2.2 Study design

This study is a prospective multicenter non-interventional longitudinal observational study.

### 2.3 Participant selection

The participants consist of all patients diagnosed with diabetes type 2 mellitus (T2DM), attending the diabetic management clinics for treatment. The eligibility criteria based on newly diagnosed diabetes (< 5 years), age > 18 years without any other systemic serious disease (e.g., arthritis, thyroid disorders, renal impairment, pregnancy, breast feeding, cancer etc), visiting primary or tertiary healthcare centers for follow-up at five different locations in Penang, Malaysia. Patients with impaired fasting blood glucose (FBS > 6 mmol/L & Hb1Ac > 6%) were eligible to participate. The FBS information was reconfirmed/proven from patient’s medical records obtained during the recruitment process from the respective sites. Patients using other prescription drugs or documented immunological disorder were excluded from the study.

### 2.4 Study duration

The study was twenty-four months (2 year) long with ten-point of assessments (baseline 0-3-6-9-12-15-18-21-24) during January 2018 –December 2019. The longitudinal non-interventional observational study design was applied with stratification to determine the effect of different diabetic medications on primary clinical outcome variables.

### 2.5 Sampling technique and enrollment procedure

**Fig 1:**
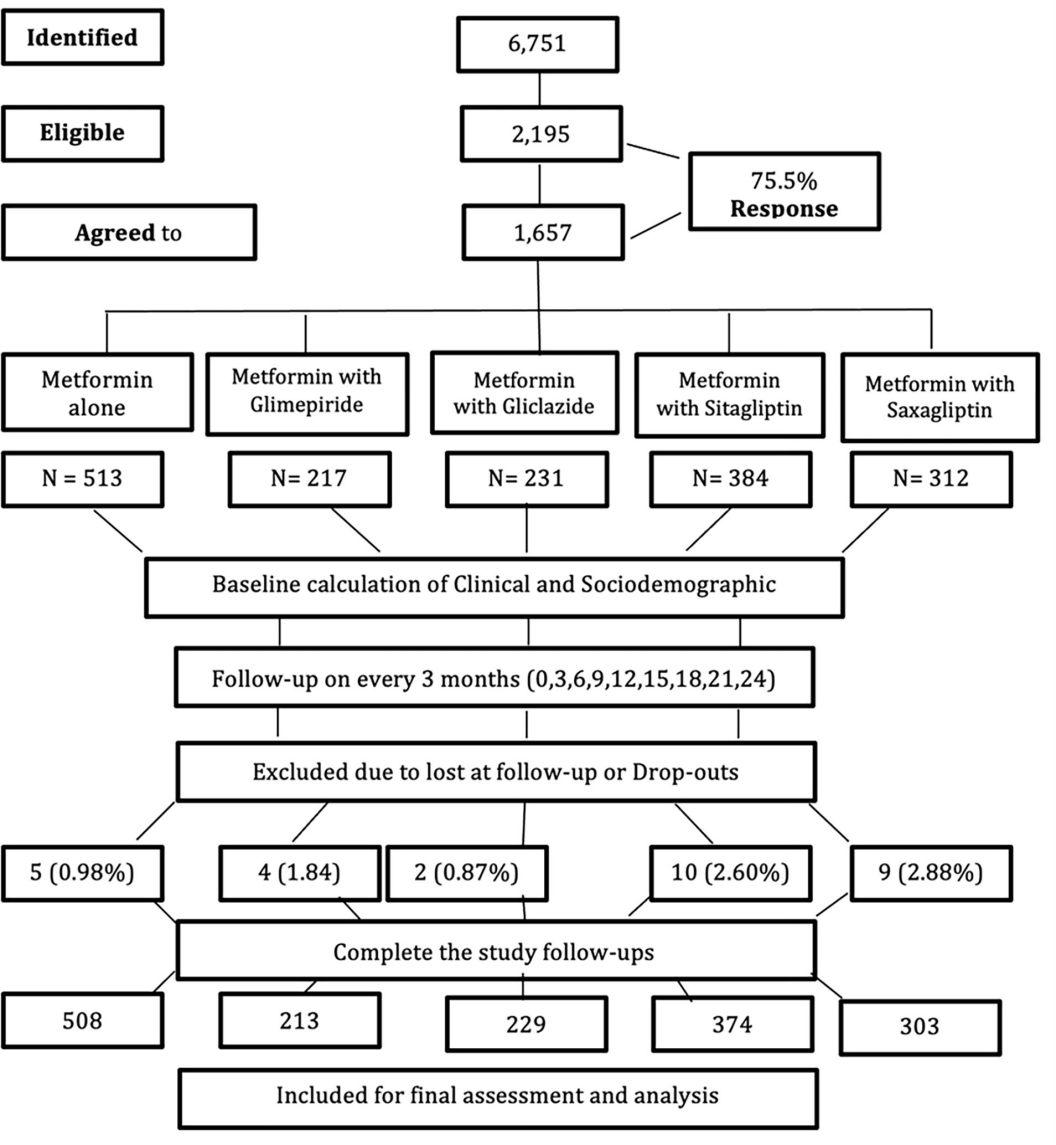
Patient recruitment and follow-up process. * G1: Metformin alone, G2: Metformin + glimepiride, G3: Metformin + glyburide, G4: Metformin + Sitagliptin, G5: Metformin + Saxagliptim.

Patients may be self-referred or recommended by physicians or healthcare professionals from the relevant sites. All the eligible patients were thoroughly screened for the inclusion/exclusion criteria. Only eligible patients were provided information with consent form and study information sheet. Research coordinator prepared documents and provide enrollment lists from all five sites to principal investigator. The procurement and cohort distribution can be seen in Fig 1.

The participants were further stratified into five groups:

- G1 (metformin alone),
- G2 (metformin with Glimepiride),
- G3 (Metformin with Gliclazide),
- G4 (metformin with Sitagliptin)
- G5 (Metformin with Saxagliptin).

During the data collection process, all study forms were labeled with a unique study identifier for each cohort. All the collected forms and data were stored in a locked file cabinet in a locked office with access to principal investigator only. Co-researcher checked for any missing or outlier values. All the participants were monitored for 24 months (2 years), and participants were required to strictly follow-up at the scheduled time at every 3 months.

At study baseline (0 month), clinical parameters including glucose profile, renal function, lipid profile and risk assessment for ASCVD [11] were calculated. Treatment protocol and care services were standard across the sites under the supervision of research team (including principal investigator). All the scheduled assessment appointments with the study participants were closely monitored by the research team in collaboration with clinicians and nurses. For quality assurance purpose all the follow-up data were reviewed and validated by principal investigator, so there was no clinical/methodological bias in the process.

### 2.6 Monitoring parameters

Body Mass index (BMI): Seca Stadiometer, as Obesity is in inclusion criteria so allowed limit = < 30kg/m^2^. Seca nonelastic tape was used to determine waist circumference (WC). Blood Pressure (BP): manual sphygmomanometer, three readings were taken 2 minutes apart (mean consider at baseline).

Fasting blood sugar: an enzymatic colorimetric method with glucose oxidase was used, required normal value < 5.6mmol/l.

Lipid profile: Total Cholesterol (Total-c), Triglycerides (TG), low-density lipoprotein cholesterol (LDL-c) and high-density lipoprotein cholesterol (HDL-c) were assessed by using commercially available kits.

ASCVD Criteria [11]: Risk score was used to assess the risk for the development of cardiovascular disease at baseline and end of the study. Individual score was calculated as per point system and calculate the mean for the whole cohort. Criteria: low risk (< 5%), Borderline risk (5% - 7.4%), Intermediate risk (7.5% - 19.9%), High Risk (> 20%).

### 2.7 Standards

BMI: Body Mass Index: (underweight: less than 18.5, healthy: 18.5–24.9, Overweight: 25–29.9).

Hb1Ac: Glycated Hemoglobin (< 6.5%), FBS: Fasting blood sugar (Normal: 4.4–7.0mmol/L, PPBS: Post-Prandial Blood Sugar, two hours after meal (Normal: 4.4–8.5mmol/L), LDL: low density lipoproteins (≤ 2.6mmol/L), HDL: High density lipoproteins (>1.0 (male) >1.2 mmol/L (female), Triglyceride (≤1.7mmol/L) and eGFR normal: 90 mL/min/1.73m^2^.

All the participants had the access of 24/7 emergency helpline: for reporting of any adverse drug reaction/event or side effect. The clinical examination was conducted for the possible reason and documented report were submitted to principal investigator for validation and recording keeping.

### 2.8 Data collection tools and achieving [12]

A trained nurse of the health care centres drew a 7ml blood sample on each visit, stored in two polyethylene-evacuated tubes for quantitative measures (FBS, lipid profile and eGFR). All the qualitative measures were performed at the respective site of recruitment. All the participants were assured of confidentiality clause in the research protocol. Regular reminders provided to each participant’s visit that they were participating on voluntarily basis and thus could decline at any time of study. All the positive efforts were added to minimize any potential bias and also to conduct this study in the most ethical manner possible.

### 2.9 Withdrawal criteria and dropout criteria [12]

Following are the withdrawal criteria used to identify dropouts and manage response among different cohorts.

- Discontinue (D/c) patient follow-ups: participant withdrew consent and/or non-cooperative.
- Participant developed condition or disease or illness that changed clinical parameters or study environment.
- Female participants became pregnant.
- Participants were clearly instructed to not take any other OTC drugs without informing the investigator at 24/7-helpline, if investigator somehow identified the use of any medications (OTC or prescribed) / herbal supplements / multivitamin supplements in any participant lead to instant D/c from the study.
- Participants missed three consecutive or alternative schedule assessments.
- Participants showed severe signs of hyperglycaemia that requires triple therapy or insulin or injectable.

**Note:** All the participants received a voice only monthly basis to ensure adequate adherence to study protocol.

### 2.10 Statistical analysis [12]

Data analysis was made using IBM SPSS Statistics, version 22 (Armok, NY). A probability of p<0.05 was considered statistically significant for all tests. Continuous variables were tested for normality; any non-normal values were categorized or transformed. All variables were analyzed using descriptive analysis. Unadjusted comparisons between study arms were made using t-tests for continuous variables or chi-square tests for discrete variables. One-way ANOVA were used to assess the difference between the groups at the baseline of randomization. Paired t-tests were used to evaluate the difference within the groups. In the intragroup analysis comparison were made between G2 Vs G3 (SU class), G4 Vs G5 (DPP4 class). To evaluate the overall clinical effect of SU and/or DPP4 class overall a longer treatment duration (2 years). Multivariate analysis was performed using the Bonferroni test. The purpose is to determine which means are significantly different, we must compare all pairs.

**Note:** Mean Weighted difference (MWD) with heterogeneity and effect z was calculated to determine the extent of effect on risk reduction score from baseline to end of the study, the study was aimed to compare the time and treatment effect among patients.

## 3. Results

### 3.1 Study participants and assessments

A total of 1,657 were enrolled to different cohorts with response rate of 75.5%. The distribution of patients was based on prescribed drug. A total of 513 (30.9%) in G1 (metformin alone), 217 (13.09%) in G2 (metformin with Glimepiride), 231 (12.85%) in G3 (Metformin with Gliclazide), 384 (23.17%) in G4 (metformin with Sitagliptin) and 312 (18.89%) in G5 (Metformin with Saxagliptin). The patients’ recruitment process and distribution pattern are provided in Fig 1. A total of 1627 (98.18%) completed the study follow-up and included for final assessment and analysis.

### 3.2 Equity and balance at baseline

The baseline characteristics are presented in Table 1. The Findings showed no significant difference among different cohorts. Slight difference among all the cohorts was seen in frequencies of comorbidities however was not significant. All the other clinical and social parameters were also non-significant.

**Table 1:** Baseline characteristics of sociodemographic and primary outcomes.

Character	G1 (n = 513)	G2 (n = 217)	G3 (n = 231)	G4 (n = 384)	G5 (n = 312)	P-value
**N(%)**						
Male	291 (56.7)	123 (56.7)	144 (62.3)	247 (64.3)	201 (64.4)	0.513
Female	222 (43.3)	94 (43.3)	87 (37.7)	137 (35.7)	111 (35.6)	
**Mean ± S.D**						
Age	36.54±1.87	35.82±3.14	36.71±2.92	37.44±2.84	36.63±2.01	0.482
BMI	24.1±2.11	25.2±1.89	23.3±1.07	24.7±2.01	23.96±2.19	0.773
** Weight(kg)**	75.1±1.39	76.4±1.77	80.1±2.91	74.4±1.81	79.3±1.62	0.551
Hb1Ac (%)	8.14±1.31	7.93±1.69	8.32±1.03	7.86±1.92	8.01±2.11	0.316
FBS (mmol/L)	8.32±2.11	8.19±2.43	8.82±2.06	8.04±2.29	7.96±1.89	0.128
PPBS (mmol/L)	9.51±1.78	9.72±1.99	9.33±2.03	9.41±1.89	9.28±1.76	0.341
LDL (mmol/L)	2.51±1.33	2.18±1.23	1.97±1.36	2.04±1.41	2.21±1.59	0.498
Triglycerides	1.63±1.01	1.70±1.21	1.51±1.39	1.68±1.82	1.74±162	0.613
eGFR (mL/min/1.73m^2^)	91.71±1.98	90.34±0.83	92.19±1.08	91.08±1.04	92.01±1.11	0.772
**Marital n(%)**						
Ever	463(90.3)	182 (83.9)	197 (85.3)	341 (88.8)	296 (94.9)	0.211
Never	50 (9.7)	35 (16.1)	34 (14.7)	43 (11.2)	16 (5.1)	
**Family History DM**						
YES	474 (92.4)	203 (93.5)	219 (94.8)	361 (94.0)	280 (89.7)	0.342
NO	39 (7.6)	14 (6.5)	12 (5.2)	23 (6.0)	32 (10.3)	
**Comorbidities**						
YES	200 (39.0)	107 (49.3)	112 (48.5)	219 (57.0)	201 (64.4)	0.051
NO	313 (61.0)	110 (50.7)	119 (51.5)	165 (43.0)	111 (35.6)	

* G1: Metformin alone, G2: Metformin + glimepiride, G3: Metformin + glyburide, G4: Metformin + Sitagliptin, G5: Metformin + Saxagliptin. BMI: Body Mass Index (underweight: less than 18.5, healthy: 18.5–24.9, Overweight: 25–29.9), Hb1Ac: Glycated Hemoglobin, FBS: Fasting blood sugar, PPBS: Post-Prandial Blood Sugar, LDL: low-density lipoproteins, eGFR: estimated Glomerular filtration rate. Normal FBS: 4.4–7.0mmol/L. Normal PPBS: 4.4–8.5mmol/L. HbA1c: ≤ 6.5%. LDL: ≤ 2.6mmol/L, HDL: >1.0 (male) >1.2 mmol/L (female). Triglyceride: ≤1.7mmol/L. eGFR normal: 90 mL/min/1.73m^2^.

### 3.3 BMI and bodyweight pattern in the study

**Fig 2:**
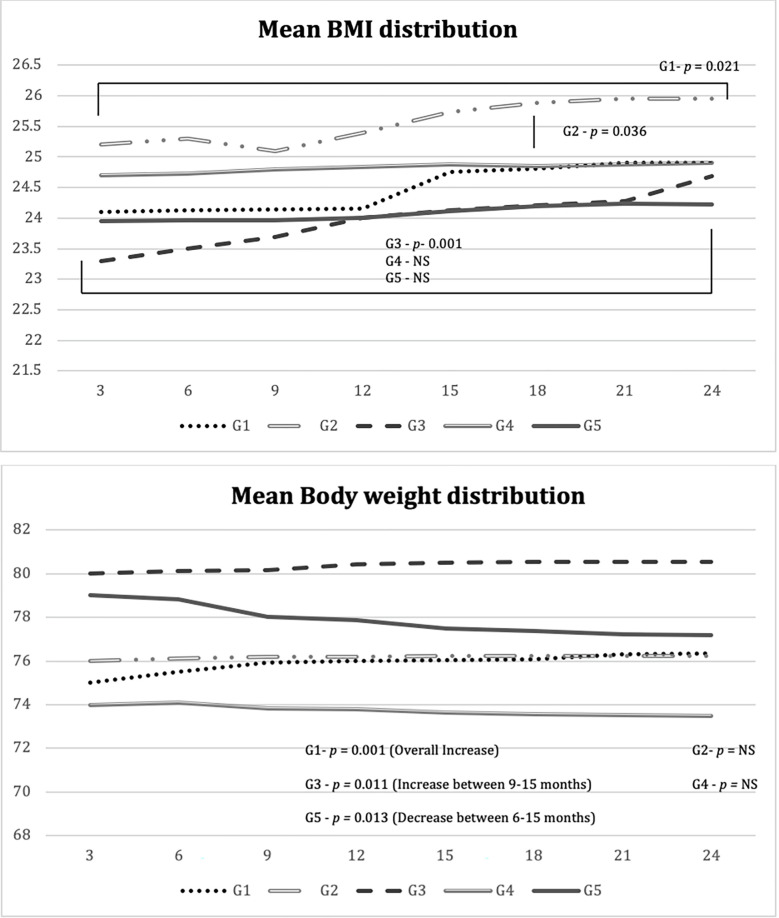
**a.** Mean distribution of BMI over 24 months of the study. **b.** Mean distribution of body weight over 24 months of the study.

The BMI distribution pattern over 24 months duration showed significant difference with Group 1 (*p =* 0.021), Group 2 (*p =* 0.036) and Group 3 (*p =* 0.001). The findings showed moderate increase in BMI among three cohorts. The analysis showed that patients with sulphonylureas (SU) have significant increase in BMI as compared to metformin alone (G1) and DPP-4 (G4 & 5). The distribution pattern of BMI over 24 months is presented in Fig 2A. In comparison to the mean body weight, a change over the 24-months showed significant difference with G1 (*p =* 0.001), G3 (*p =* 0.031 and G5 (*p =* 0.001). The increment in the mean bodyweight was seen in G1 however a significant reduction was seen in G5 (*p* = 0.001) between 6–15 months of the study duration. The details are provided in Fig 2B.

### 3.4 Within group assessments of primary outcomes

The Intergroup analysis showed significant differences with all the primary outcome variables except BMI (*p* = 0.217) and eGFR (*p =* 0.782) among patients using sulphonylurea (SU) combination (G2 & G3). Low-density lipoproteins were the only primary variable that was not significant (*p =* 0.431) in the intergroup assessment of patients using DPP-4 combination (G4 & G5). Intra-group analysis showed significant differences in mean difference of primary outcome variables between control group (G1-metformin alone) compared to SU and DPP-4 combinations. All the detail analysis is provided in Table 2. Findings also showed significant high frequency of emergency visit and hospitalization due to more episodes of hyperglycemia in G1 (78.16% & 30.8%) as compared to SU (70.1% & 28.3%, *p =* 0.001) and DPP-4 (56.6% & 20.4%, *p =* 0.001). The Intra-group analysis between SU & DPP-4 combination reported significant difference (*p =* 0.001) in frequency of gastrointestinal disturbance and lethargy as reported adverse drug reaction. The detailed analysis and reported significant values are provided in Table 2.

**Table 2:** Differential data analysis for the groups in the study (post 24 months).

Character	Metformin alone (n = 513)		Metformin + SU (n = 448)		*p* ǂ	Metformin + DPP4 (n = 696)		*p* ǂ	SU Vs DPP-4
	value	*p*-value	value	*p*- IG⌃		value	*p*- IG⌃		*p*-value≠
**N (%)**									
Male	291 (56.7)	0.441	267 (59.5)	0.121	0.317	448 (64.36)	0.021	0.001	0.001
Female	222 (43.3)		181 (40.4)			248 (35.63)			
**Mean ± S.D**									
BMI	24.91±1.81	**-**	25.39±1.43	0.217	0.001	24.52±1.77	0.022	0.343	0.001
Hb1Ac (%)	7.34±1.07	**-**	6.73±2.65	0.019	0.001	6.22±2.07	0.001	0.000	0.022
FBS	7.01±2.44	**-**	6.58±2.87	0.012	0.001	6.43±2.51	0.001	0.519	0.001
PPBS	8.32±1.09	**-**	8.49±1.65	0.024	0.432	8.32±1.21	0.001	0.001	0.028
LDL	2.43±0.88	**-**	1.93±0.79	0.001	0.001	1.88±0.65	0.431	0.001	0.014
Triglycerides	1.52±0.43	**-**	1.34± 0.31	0.001	0.021	1.19± 0.47	0.001	0.001	0.031
eGFR	83.42±1.62	**-**	92.32± 2.94	0.782	0.001	89.92±2.27	0.041	0.001	0.017
**Emergency visits N(%)**	401 (78.16)	-	314 (70.1)	-	0.001	394 (56.61)	-	0.001	0.001
Hospitalization * N(%)	158 (30.8)	-	127 (28.3)	-	0.034	142 (20.4)	-	0.001	0.021
**ADRs Reporting**^**ᴓ**^ **N(%)**									
Hypoglycemia	112 (21.8)	-	294 (65.6)	-	0.001	259 (37.2)	-	0.001	0.033
GI Disturbance	98 (19.1)	-	79 (17.6)	-	0.001	194 (27.9)	-	0.001	0.001
Loss of appetite	159 (30.99)	-	189 (42.2)	-	0.321	178 (25.6)	-	0.038	0.651
Weight gain	188 (36.6)	-	31 (6.92)	-	0.001	15 (2.2)	-	0.011	0.035
Lethargy	282 (54.97)	-	-	-	0.001	118 (16.9)	-	0.001	0.001
HBP	-	-	22 (4.91)	-	0.031	17 (2.44)	-	0.544	0.569
LBP	161 (31.4)	-	209 (46.6)	-	0.041	211 (30.3)	-	0.679	0.614

**⌃**IG: Intergroup *p-*value. SU: Sulphonylureas (both G2 & G3), DPP-4: Dipeptidyl peptidase 4 (both G4 & G5).

ǂ comparison with Metformin alone (G1).

≠ comparison of control G1 with SU & DPP-4 groups.

* Hospitalization primary to diabetes (hyperglycemia etc.).

^ᴓ^ Adverse drug reactions (ADRs) reported by patients during 2 years of study follow-up. GI: Gastrointestinal. HBT: High Blood pressure, LBP: Low Blood pressure. Normal FBS: 4.4–7.0mmol/L. Normal PPBS: 4.4–8.5mmol/L. HbA1c: ≤ 6.5%. LDL: ≤ 2.6mmol/L, HDL: >1.0 (male) >1.2 mmol/L (female). Triglyceride: ≤1.7mmol/L. eGFR normal: 90 mL/min/1.73m^2^.

### 3.5 Cardiovascular risk reduction pattern in different cohorts

The inter and intra-group analysis were also required to determine the effect of medication on the cardiovascular risk. The ASCVD risk assessment method was used to determine the difference between from baseline to endpoint. The findings showed signification mean reduction -1.1% (MWD: -1.02, 95%CI: -1.69 to -0.89, *p =* 0.041) in ASCVD risk score among patients using SU combination. Similarly, significant mean reduction -1.56% (MWD: -1.62, 95%CI: -2.18 to -1.02, *p =* 0.001) in ASCVD risk score was found among patients on DPP-4 combination. The overall reported effect was z = 2.58, *p* = 0.001. Table 3 presented detailed pre-post analysis of ASCVD risk.

**Table 3:** ASCVD risk assessment and comparison between different cohorts.

Cohorts	Weightage %	*p-* value	Pre (0 month)	Post (24 month)	Mean % reduction	MWD
			mean±S.D	Mean±S.D		(95% CI)
G1 –metformin (n = 513)	30.9%	0.077	6.32 ± 12.84	5.94 ± 10.11	- 0.36	- 0.31 (-0.43 to—0.07)
G2 + G3 (SU) (n = 448)	27.0%	0.041	6.41 ± 11.23	5.31 ± 9.83	- 1.1	- 1.02 (- 1.69 to– 0.81)
G4 + G5 (DPP-4) (n = 696)	42.0%	0.001	6.65 ± 11.84	5.09 ± 10.19	- 1.56	- 1.62 (- 2.18 to– 1.02)

SU: Sulphonylureas (both G2 & G3), DPP-4: Dipeptidyl peptidase 4 inhibitor (both G4 & G5).

ASVD: low risk (< 5%), Borderline risk (5% - 7.4%), Intermediate risk (7.5% - 19.9%), High Risk (> 20%).

MWD: Mean weighted difference, Heterogeneity = Chi^2^ = 0.29, df = 2 (*p =* 0.04); I^2^ = 32%

Test for overall effect = z = 2.58 (*p =* 0.001).

## 4. Discussion

Patients with diabetes mellitus have deranged lipid profiles [12]. The use of antidiabetic medications has been associated to improved lipid profiles [13, 14]. This study reported that patients on both sulfonylureas and DPP 4 inhibitors-based combinations with metformin had improvements lipid profiles especially triglycerides and LDL over 24 months compared to baseline. Among both groups, the participants with the use of sulfonylureas had greater improvement over DPP 4 inhibitors. Our findings contrast with the findings of Kim *et al* 2013 and Nomoto *et al*, who found that the use of sulfonylureas led to significant, increase in LDL [15, 16]. We also found significant decrease in triglycerides in patients receiving sulfonylureas and DPP 4 inhibitors in combination metformin, which is different from the reported literature [15–17]. These contrast findings might be the difference in geographical location and life-style modification in the region.

There has been an improvement in the glycemic control among patients in all the study groups. Though HbA1c improved among all, a greater reduction in HbA1c from baseline was seen in patients treated with DPP4-metformin combination compared to SU-metformin showing a similar pattern of decrease as seen in studies conducted by Nomoto *et al* and Goke *et al*. [16, 18]. In contrast, there are some studies reported SU-metformin combination decreased HbA1c more than DPP-metformin combination [19, 20].

Impact on glycemic parameter (FBS), the results of our study have shown that the patients receiving SU-metformin combination had better control compared to DPP-metformin combination showing a similar pattern of results compared to others in literature [21]. In contrast, the aspect of post prandial control of glycemic control, DPP-metformin combination use led to better outcome compared to SU-metformin, similar findings are reported by Sharma *et al*. [22].

Presence of obesity and overweight increases the risk of insulin resistance in diabetic patients. Hypoglycemic agents are associated with weight gain. Our study found that patients treated with sulfonylureas, there was an increase in body weight and BMI, as found in most of the scientific literature [20, 23]. The other important finding of the study was a significant decrease in body weight and BMI among patients treated with DDP4 inhibitor-metformin combination and consistent with the findings of previous studies [17, 18, 20]. However, this study also identifies the efficacy mnemonics of different drugs in DPP4 and SU class.

Yet DPP-4 inhibitors have a better safety profile compared to sulfonylureas. Majority of the study population who developed hypoglycemia were belongs to sulfonylureas group compared to DPP 4 inhibitors, similar findings are reported in the literature [17, 23–25]. However, gastrointestinal disturbances and lethargy was seen more among patients treated with DPP4 inhibitors, showing similar findings as observed by Kim *et al*. [17]. In-group analysis of this study provided in-depth knowledge of drug mnemonics and toxicity profiling.

Patients on antidiabetic medications often reported hospital emergency visits leading to hospitalizations in some instances. It is difficult to predict the risk of hospital visits among different types of drugs. However, this study attempted to determine the number of emergency visits and hospitalizations among different groups treatment with different combinations. It was found that patients using DDP4 inhibitor-metformin compared to the sulfonylurea-metformin combination reported lower hospital visits. The reason for these findings might be due to reduced incidence of hypoglycemia with DDP4 inhibitor and their improved glycemic control leading to fewer risks of emergency conditions like diabetic ketoacidosis [24, 26–29]. Combination of SU or DPP-4 inhibitors were prescribed more to the patients with worse comorbidities than control group (G1) metformin only. Yet, combination treatment had better outcome or improved cardiac risks. Among the sulfonylureas, Gliclazide was shown to have better cardiovascular profile [30, 31]. SAIS1 trial reported that treatments with DPP4 inhibitors have favorable effects on inflammatory mediators and oxidative stress in patients with T2DM without advanced atherosclerosis [16]. The cardiovascular risk (ASCVD) assessed and comparison among different combination of antidiabetic medications was another significant and novel finding of this study. It was found that ASCVD risk score was reduced in both groups receiving sulfonylureas or DPP- 4 inhibitor-based combination. In a recent literature also, it was reported that DDP4 inhibitor and sulfonylureas drugs lead to greater reduction in risk of CVD compared to metformin alone [21, 23].

## 5. Conclusion

The study findings concluded the effective role of sulfonylurea combination in reduction of LDL and triglycerides among patients with type 2 diabetes mellitus and not known serious clinical comorbidities. The study also concluded significant effect of *Dipeptidyl peptidase*-4 inhibitor on reduction of hospitalization, lipid profile and also ASCVD risk score of type-II diabetes mellitus patients regardless of clinical comorbidities. The distribution pattern suggested significant changes in primary clinical variables during first 6–15 months of therapy. Clinicians and healthcare professionals should be proactive in the management of secondary clinical sign & symptoms to maintain patient compliance and adherence.

## 6. Limitations of the study

As of all other studies this study also reported few limitations as follow.

- Patients’ censorship: change of medication during the study leads to censorship and thus the secondary analysis is required to identify the effect and confounding variables relative to change in regimen.
- Lack of generalizability to Type 2 diabetic patients with other comorbidities.
- Study didn’t apply equal randomization among all participating sites.
- Pharmacoeconomic impact of combination drugs. Cost-effective might have a influence on the prescribing practices.

## Abbreviations

DM: Diabetes Mellitus
T2DM: Type 2 Diabetes Mellitus
DPP-4: *Dipeptidyl peptidase*-*4* inhibitors
SU: Sulphonylurea
ASCVD: Atherosclerosis cardiovascular disease
BMI: Body Mass index
Hb1Ac: Glycated Hemoglobin
FBS: Fasting Blood sugar
PPBS: Post-prandial blood sugar
LDL: low-density lipoprotein
eGFR: estimated Glomerular-filtration rate
GI: Gastro-intestinal
HBP: High blood pressure
LBP: Low blood pressure
MWD: Mean weighted difference
